# Deep learning approaches for diagnosing seizure based on EEG signal analysis

**DOI:** 10.3389/fnhum.2025.1669919

**Published:** 2025-11-10

**Authors:** Mohammed Alarfaj, Muhammad Ali Zeb, Mosleh Hmoud Al-Adhaileh, Asma Abdulmana Alhamadi, Nadhem Ebrahim

**Affiliations:** 1 King Salman Center for Disability Research, Riyadh, Saudi Arabia; 2 Department of Electrical Engineering, College of Engineering, King Faisal University, Al-Ahsa, Saudi Arabia; 3 Institute of Computing, Kohat University of Science and Technology, Kohat, Pakistan; 4 Deanship of E-Learning and Information Technology, King Faisal University, Al-Ahsa, Saudi Arabia; 5 Department of Humanities, College of Science and Theoretical Studies, Saudi Electronic University, Riyadh, Saudi Arabia; 6 Department of Computer Science, College of Engineering and Polymer Science, University of Akron, Akron, OH, United States

**Keywords:** personalized seizure detection, transfer learning, EEG signal analysis, deep learning, patient-specific models

## Abstract

**Introduction:**

Epilepsy is diagnosed in about 1% of the world’s population as a common brain disease. Timely prediction and detection of seizures can significantly improve the lives of epilepsy patients.

**Methods:**

The study has garnered considerable attention over recent years, particularly in the context of advanced computational methods. However, current seizure detection methods still face several limitations, including high inter-patient variability, noisy and non-stationary EEG signals, and the limited generalization ability of single deep learning (DL) models. This paper presents an Ensemble of Deep Transfer Learning (EDTL) models for personalized seizure detection. The technique combines ResNet and EfficientNet methods along with a customized two-Dimensional Convolutional Neural Network (2DCNN) method for patient-specific seizure detection using EEG data. Raw data from the recordings of seizure patients is transformed into EEG signals. Personalized sliding windows are used to extract and store spectrograms for the patients. Patient-specific features are extracted from individual records. EEG signals are normalized for consistent scaling. Short Time Fourier Transform (STFT) is then applied for continuous window slicing over short time intervals. To address the limitations above, the proposed EDTL framework integrates general-purpose pre trained models with a domain-specific custom 2DCNN to capture complementary features. This design improves robustness against noise, enhances adaptability to patient-specific variability, and achieves better generalization compared to individual models. The transformed data is then passed on to train and optimize the models independently and later combined into EDTL.

**Results and discussion:**

A comparative evaluation is performed using standard evaluation metrics on two datasets, the CHB-MIT Scalp EEG Database and Turkish Epilepsy EEG Dataset. The proposed EDTL models are evaluated against the individual models on standard performance metrics, with the EDTL achieving the highest performance of 99.23% on the AUC.

## 1 Introduction

Epileptic seizures are caused by abnormal electrical activity in the brain and can severely affect a person’s health and quality of life. Early detection is critical for preventing serious complications. Electroencephalogram (EEG) signals are widely used for seizure detection due to their ability to capture brain activity in real time. However, seizure patterns vary greatly across individuals, making it difficult to design a single model that works well for all patients (Amin et al., 2020). This inter-patient variability arises from variations in brain shape, seizure patterns, and signal characteristics, necessitating a customized approach for seizure detection. Epilepsy, a prevalent neurological disorder that impacts individuals across all age groups, can be fatal if not detected timely and left untreated. A seizure is a neurological condition characterized by a complex chemical change in brain nerve cells, generating electrical signals (Amin et al., 2020). This can lead to mild jerks, severe convulsions, and impaired movement, bowel and bladder control, and cognitive functions. It also disrupts consciousness and cognitive functions (Sharmila and Geethanjali, 2016). Epilepsy affects 70% of adults and 30% of children, with 70% etiology unknown. Recurrent seizures are classified as partial or generalized, with one type being partial (Sharmila and Geethanjali, 2019).

To address the complexity of EEG signals, DL methods have been widely explored. RNNs, especially LSTM and GRU networks (Shekokar and Dour, 2022; Chauhan and Vig, 2015), are popular for modeling sequential EEG data. While GRUs are faster and lighter, LSTMs are more accurate with long sequences, making them preferred in many studies. CNNs are often combined with LSTM or GRU to capture the temporal features of EEG along with the spatial features. However, standard encoder-decoder LSTM models may lose important information due to compression into a single vector. Attention mechanisms have been introduced to solve this issue by helping the models to prioritize more significant segments of the EEG signals (Guo et al., 2020).

The typical morphology of EEG data is altered during an epileptic seizure. Consequently, three phases can be identified for the state categorization of epileptic patients, based on the diverse properties of EEG signals. These three phases are normal, preictal, and ictal. Prior to the actual initiation of a seizure, numerous electrical abnormalities start in the cerebrum of epileptic patients. This is referred to as the preictal period. To identify seizures at this time, it is essential to document the electrical abnormalities in the patient’s brain during the shift from normal to the ictal phase (Kulaseharan et al., 2019; Zazzaro et al., 2021; Van Klink et al., 2019; Fountas and Kapsalaki, 2019; Subasi et al., 2019; Acharya et al., 2018; Lauretani et al., 2021; Carbó-Carreté et al., 2020; Morales Chacón et al., 2021; Takagi et al., 2020). Thus, it is imperative to identify epileptic seizures early, in the preictal stage. The lives of the patients can possibly be saved by allowing the practitioners to implement preventative measures in a timely manner to avert harmful and perhaps fatal incidents. In EEG examinations, the brain’s electrical activity is detected by electrodes. A paste like medium or cap is used to affix these electrodes to the scalp.

EEG signal patterns change significantly during a seizure, typically progressing through preictal, ictal, and postictal phases. Detecting seizures in the preictal phase is vital, as it allows preventive action. To record the EEG signals, electrodes are placed on the scalp, which makes real-time, non-invasive monitoring possible. Traditional seizure detection relies on handcrafted features and large labeled datasets, which can be time-consuming and hard to generalize. Training DL from scratch for each patient is also not practical. Transfer Learning (TL) offers a better approach by adapting pre trained models to new tasks using smaller data. This helps build faster, more accurate seizure detection models tailored to individual patients.

Traditional seizure detection strategies frequently rely upon vast classified records and handmade functions that are hard work-in-depth and can fail to generalize across patients (Sharmila and Geethanjali, 2016). DL has demonstrated considerable promise in automating function extraction and improving detection accuracy; however, training a DL model from scratch for each patient is resource-intensive and impractical. TL, which permits the model of pre-trained fashions to new responsibilities or domains with limited statistics, offers a feasible option to cope with this mission (Sharmila and Geethanjali, 2019; Kulaseharan et al., 2019). By leveraging expertise from a regularly occurring base version trained on a big dataset, TL can create efficient and optimized models for individual patients, achieving better accuracy and efficiency in seizure detection.

In this paper, we propose an ensemble of pertained DL models combined with patient-specific optimization, called EDTL. This model adapts general knowledge from existing models to each patient’s data, improving detection performance with fewer resources. We chose commonly used DL models (ResNet, EfficientNet, and a custom CNN) for comparison to show the benefits of transfer learning in seizure detection. While these may not reflect the most recent SOTA models, they are widely accepted in literature and provide a solid baseline. Future work will extend this study by comparing EDTL with more advanced, SOTA approaches on larger datasets.

The contributions of this work are:

1. A comprehensive, computationally efficient framework for personalized seizure detection.
2. Analysis of pre trained models, together with transfer learning and domain-specific optimization.
3. Empirical evaluation of the proposed EDTL approach, demonstrating its advantages using standard evaluation protocols.

The paper is organized as follows: Section 2 offers a review of the existing literature on seizure detection techniques specifically and the applications of transfer learning in the healthcare domain generically. Proposed materials and methods are delineated in Section 3, encompassing aspects such as dataset preparation, model architecture, and transfer learning strategies. Section 4 outlines the experimental framework and presents the results, emphasizing the performance of personalized models in comparison to baseline methods. In Section 5, the paper is concluded by presenting a summary of the principal findings, a discussion of the potential limitations, as well as suggesting potential possibilities for future research.

## 2 Literature review

Most automated seizure detection systems employ ML and DL techniques that consist primarily of two processes: feature engineering and classification (Boonyakitanont et al., 2020; Anusha et al., 2012; Adeli et al., 2007). The non-stationary characteristics of EEG signals necessitate considerable effort and specialized knowledge in the feature extraction process to analyze and assess the signals (Farooq et al., 2023; Siddiqui et al., 2020). An important concept to consider is the automatic learning and extraction of features directly from raw data, independent of human expertise. Manasvi Bhat et al. (2019) conducted an analysis utilizing actual data sourced from the Epilepsy Ecosystem. Various signal processing techniques and mathematical operations are utilized for extracting features from the data following preprocessing. Models are developed utilizing diverse combinations of these characteristics alongside supervised learning methods such as XG Boost and Extra Trees Classifier, applicable to both generalized and patient-specific contexts. These models are designed to endure noise and maintain robustness. It is observed that the generalized model utilizing XG Boost, trained with enduring features, attains a higher level of accuracy.

Almustafa (2020) identify a correlation between seizures and abnormal brain activity associated with epilepsy, characterized by a range of symptoms. Furthermore, dataset prediction employing feature selection based on attribute variance was performed. The dataset on epileptic seizures was classified using various methods. Further analysis examined various factors, including the divisions of the training and testing sets in the random forest, as well as the learning rate, regularization parameter, and loss function of the stochastic gradient descent (SGD). The findings indicate that enhancing classification accuracy is attainable through the fine-tuning of specific classifier parameters. Researchers have vastly utilized Machine learning (ML) algorithms for the identification of seizures in newborns. Purnima and Kattepura (2023) have explored ML algorithms for detecting neonatal seizures. The utilization of ML to tackle this challenge is promising, as early seizure prediction may enable implanted neuro stimulators to intervene and avert seizures. The study employs machine learning and DL methodologies to forecast epileptic episodes. ML-based architecture is presented by the study that exhibits optimal performance on prediction, like earlier models, while requiring minimal configuration. The study evaluates various methods for seizure prediction using EEG and various ML models, including advanced techniques. The proposed classifier is trained on a publicly available dataset of NICU seizures recorded at Helsinki University Hospital and evaluated utilizing standard evaluation methods.

Amin et al. (2020) introduced an innovative approach utilizing ML techniques for the automatic identification and diagnosis of epileptic episodes in EEG recordings. The author identifies and categorizes characteristics through wavelet analysis and arithmetic coding. The text examines the challenges and opportunities associated with predicting epileptic seizures through the application of ML techniques. This article offers insights into the identification of gaps and challenges in accurate seizure detection. Furthermore, it proposes potential avenues for future research in this domain. The research methodology included a thorough literature review, focusing on the selection process of pertinent papers and the use of abstract-based key wording to determine the most relevant keywords. The study presents a classification that encapsulates the advanced solutions for the issue (Walther et al., 2023). This study introduces a new architecture that employs deep recurrent neural networks (DRNN) for the automated identification of patient-specific seizures using scalp EEG data. Furthermore, the objective is to map seizure EEG signals to facilitate efficient processing using the DRNN. This mapping allows the DRNN architecture to concurrently learn the spatial as well as the temporal characteristics of raw seizure EEG signals. DRNN architecture is evaluated using long-term scalp EEG data from five subjects, amounting to approximately 34 h, sourced from a publicly available dataset. The proposed network effectively identifies all seizure occurrences, exhibiting an average detection delay of 7.0 s. Statsenko et al. (2023) examine the potential benefits and challenges associated with utilizing this data to improve seizure detection, ultimately aiming to enhance the quality of life for patients. Additionally, the author examines the application of ML in analyzing and extracting features from EEG signals, presenting methods to attain high classification accuracy.

Omar et al. (2024) presented a distinctive classification method for EEG time series utilizing RNNs that incorporates LSTM networks. Their proposed deep network effectively extracts and visualizes distinct temporal patterns from sequential EEG data. Features are derived automatically from unprocessed raw EEG data, eliminating the necessity for preliminary processing and reducing the manual effort involved in feature construction. Asif et al. (2020) present a DL approach utilizing a dense CNN to train robust features across various temporal and spatial EEG data spectrum resolutions. This enables precise classification of seizure types among patients. In Xu et al. (2020), have analyzed EEG data for the automatic identification of epileptic seizures, proposing the 1D CNN-LSTM model. The analysis is performed by initially preprocessing and normalizing the raw EEG signal data. The normalized EEG sequence data is utilized to construct a 1D CNN, which efficiently extracts information. Lebal et al. (2023) introduced a collection of DL tools, Epilepsy-Net, designed for the processing of EEG signals, with the objective of distinguishing between epileptic and non-epileptic seizures. The Epilepsy-Net framework integrates various components, including 1D-CNNs, RNNs, and attention mechanisms. Specific models instantiate each algorithm: the convolutional block attention module for attention mechanisms, gated recurrent units for RNNs, and ResNet and Inception for CNNs. However, the author validated Epilepsy-Net through the analysis of multiple extensive public EEG signal datasets. The experimental results demonstrate that the attention-based DL technique is highly effective in accurately detecting epilepsy from EEG signals. Riyazulla Rahman (2023) has discussed in detail the application of advanced computational methods for the automation of the detection of epileptic seizures, specifically focusing on DL techniques. The performance of various DL architectures, including SeizNet, 2D-CNN, and 1D-CNN, was highlighted. Geethanjali (2015) have utilized binary classification to propose a method that divides the EEG signal activity into seizure and non-seizure classes. The method has been shown to efficiently differentiate between the two classes. The k-NN classifier is used to execute multiple classification tasks. Mirjalili et al. (2014) propose several features that improve the efficacy of the seizure prediction model. Significant evaluations are observed in learning methodologies involving RNNs, CNNs, and SVM. They have implemented a multitude of kernel functions for improving the predictive performance of the classification models. Another study Moldovan (2022) employed an RNN model for the recognition of epileptic seizures via binary classification. An RNN model was employed for the classification of monitored data, utilizing LSTM for the first layer and the Horse Optimization Algorithm (HOA) for the dropout layer. Manocha et al. (2022) employed a one-dimensional CNN for classifying EEG time series data included a one-dimensional CNN module combined with a ResNet module to determine the presence of epilepsy, resulting in an AUC of 98%. Nanthini et al. (2022) utilized an LSTM model to train, detect, and predict epileptic seizures, including state changes and chaotic EEG seizures. This research aimed at developing a small, low-cost wearable device.

Deep transfer learning has been vastly studied for disease prediction (Shaikh et al., 2025; Khaliki and Başarslan, 2024) generally and seizure prediction specifically (Lopes et al., 2024; Wei and Mooney, 2023). Similarly, an ensemble of deep machine learning models has also been successfully applied for the prediction of different diseases (Shaikh et al., 2025; Mou et al., 2024; Reshan et al., 2023; Saleh Al Reshan et al., 2024). He et al. (2024) have proposed SeizureLSTM, where the raw EEG signals are decomposed into different frequency bands by utilizing Tunable Q Wavelet Transform. These are subsequently used for extracting the informative signal features, using 1DCNN and spectral features. The work relates to seizure detection using an optimized LSTM-based deep model with attention. Yuan et al. (2024) presented a hybrid model for seizure prediction combining DenseNet architecture for fine-grained spatial feature extraction and Vision Transformer (ViT) architecture for global context modeling, with an attention fusion layer to adaptively combine their outputs. They leverage the efficiency of DenseNet for capturing hierarchical features, while self-attention mechanisms of ViT are utilized for global feature representation. The methodology involves preprocessing raw EEG signals using STFT to create time-frequency matrices, which are then processed through the hybrid network for seizure prediction. The researchers evaluated their model using the CHB-MIT dataset, employing leave-one-out cross-validation for performance assessment. The approach is related to the proposed work since it uses EEG with DenseNet and attention mechanism, both often leveraged in seizure-related neural decoding. Apart from the work specifically related to seizure detection, the field can also benefit from the methods applied in relevant disciplines. The Reseek-Arrhythmia model Yang et al. (2024) uses DL techniques for automatic heart arrhythmia detection and classification. The model leverages ResNet architectures and transfer learning approaches, making it relevant for similar signal processing challenges in medical diagnostics. The model focuses on arrhythmia detection using DL architectures (ResNet), which is highly relevant as arrhythmia and seizure detection share signal processing and classification challenges. Transfer learning approaches are often employed in arrhythmia detection, making this a directly relevant comparison (Yang et al., 2025).

According to our knowledge, many existing methods for seizure detection use machine learning or DL with manually selected features. While models like CNNs and LSTMs perform well, they often require a lot of expert knowledge and do not work equally well for all patients. After reviewing the literature review, it can be observed that some recent studies use transfer learning and attention-based models, but most still lack proper personalization and are not efficient enough for real-time use.

The proposed research offers a simple and efficient approach that combines pre trained models, transfer learning, and tuning for specific patients. It helps improve accuracy and speed while adapting to each person’s data. This makes the system more flexible and useful in real-world healthcare settings. Unlike earlier models, the proposed framework learns directly from the data with less manual effort. It also supports faster deployment across different users, making it suitable for clinical use.

## 3 Materials and methods

**Figure 1:**
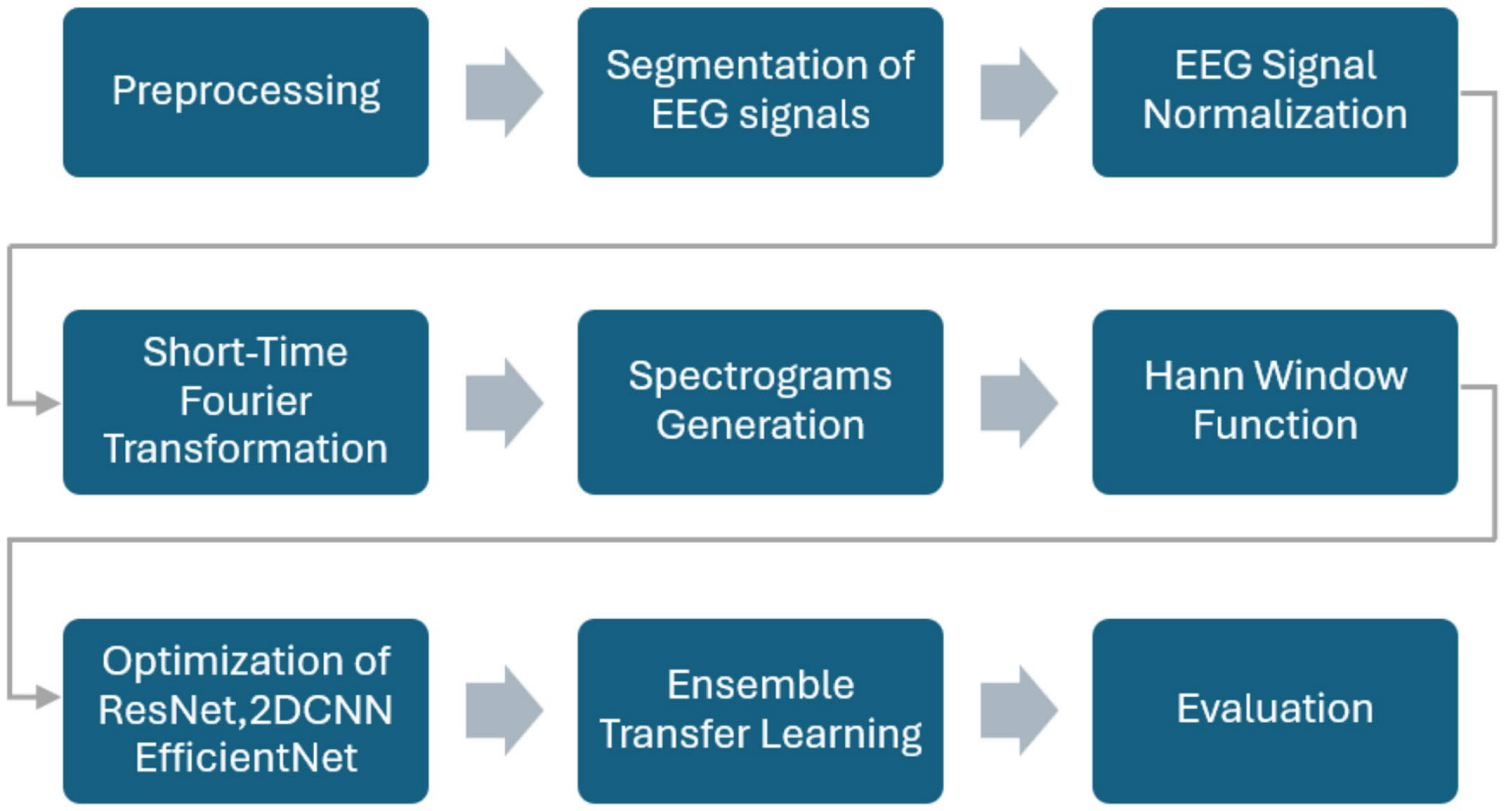
Framework of the proposed methodology.

The proposed methodology uses an ensemble of optimized DL algorithms using transfer learning for personalized seizure detection. The publicly available CHB-MIT database is employed to train and optimize the models separately and subsequently combined to form an ensemble of the models. The dataset is explained in detail in the experiments section. Figure 1 exhibits the step-by-step process of the methods and materials used in the work. Raw EEG signals are preprocessed, normalized, and segmented. The STFT is applied to generate overlapping spectrogram windows. The spectrograms are then used for feature extraction and fed into pre-trained models (ResNet-18, EfficientNet-B0, and a 2DCNN). The models are optimized individually, combined into an ensemble, and finally evaluated using standard performance metrics.

### 3.1 Preprocessing and segmentation

Preprocessing is performed to extract the EEG signals from the recordings of seizure patients. Personalized sliding windows are used to extract and store spectrograms for the patients. Patient-specific features are extracted from individual records. EEG signals are normalized for consistent scaling.

### 3.2 EEG signal normalization

EEG signals were normalized for consistent scaling across patients and recording sessions. Normalization ensured that variations in amplitude due to electrode placement or recording conditions did not bias the model training process.

### 3.3 STFT method

STFT is then applied for continuous window slicing over short time intervals. STFT is used for avoiding the loss of information in the form of spectral leakage by generating overlapping spectrogram windows. In STFT, a signal is broken into overlapping segments, the Fourier Transform is applied to each segment, and the results are combined. For a given discrete signal s[n] , the STFT is defined in Equation 1 as:


$$X(t,k)=\sum_{n=0}^{N-1}x[n]w[n-\mathrm{tR}]e^{-j2\mathrm{\pi kn}/N}$$ (1)

where X(t,k) is the result obtained at time index *t* and frequency index *k*, x[n] is the input signal, w[n] is the windowing function for the current sample index *n*, *R* is the step size overlap between the segments, and *N* is the length of the segment. A spectrogram is then computed as the squared magnitude of the STFT S(t,k)=∣X(t,k)∣2 . We have chosen a window size of 8 s with a step size of 4.

### 3.4 Spectrogram generation

**Figure 2:**
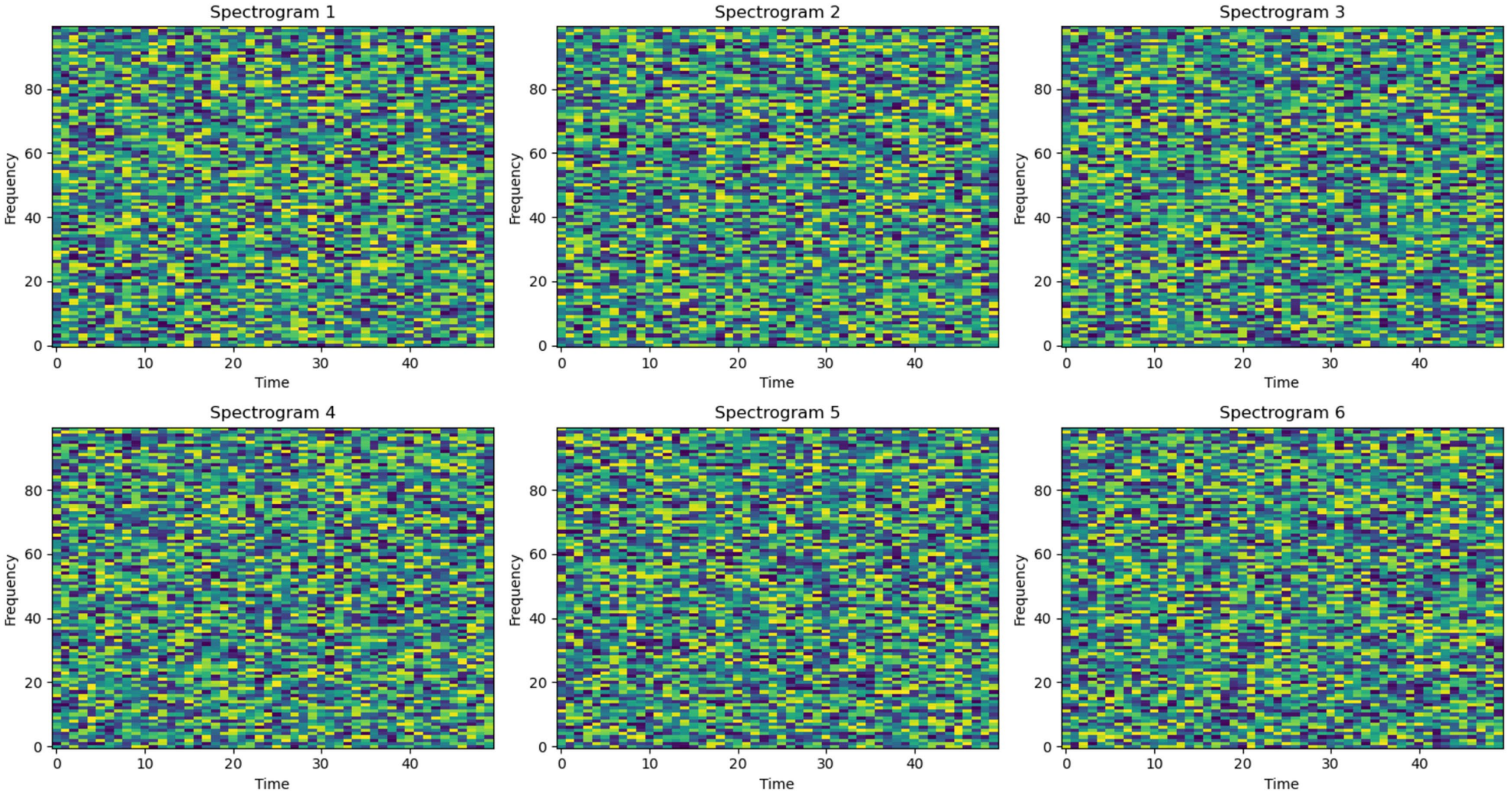
Spectrograms.

To further reduce the noise, spectrograms are generated from the EEG signals. This step improves the performance of the individual as well as the ensemble of the models. Figure 2 shows the sample spectrograms extracted.

### 3.5 Hann window function

The Hann Windowing function (Pielawski and Wählby, 2020) w[n] used be defined in Equation 2 as


$$w[n]=\frac{1}{2}(1-\cos(\frac{2\mathrm{\pi n}}{N-1})),0\leq n\leq N-1$$ (2)

The windowed signal $x_{w}[n]$ is then obtained using the product of the original input signal x[n] and the Han window w[n] , i.e., $x_{w}[n]=x[n].w[n]$ . The Hann Window function minimizes the spectral leakage by smoothly tapering the signal to zero at the edges, minimizing the effect of sharp discontinuities. The spectrograms and the labeled windows are then passed to the models.

**Figure 3:**
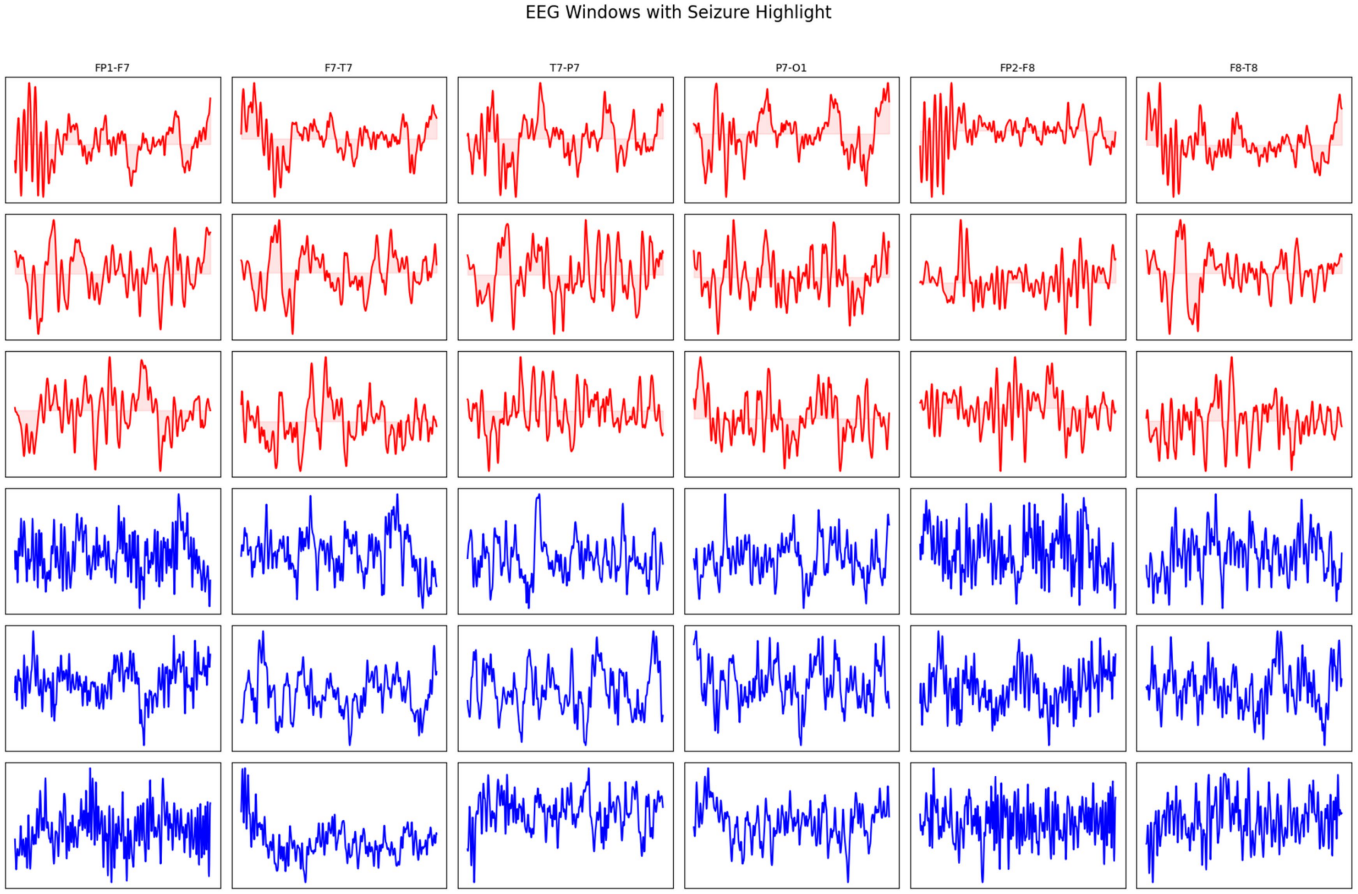
EEG windows showing seizure (red) and non-seizure (blue) activity across different channels.

In addition, EEG windows with seizure and non-seizure activity are highlighted across multiple channels in Figure 3. Red traces represent seizure activity, characterized by abnormal and synchronized electrical discharges, while blue traces represent non-seizure (normal) activity. This visualization provides a comparative overview of the temporal and spatial differences in brain signal patterns between seizure and non-seizure states.

### 3.6 Proposed 2-phase model

**Figure 4:**
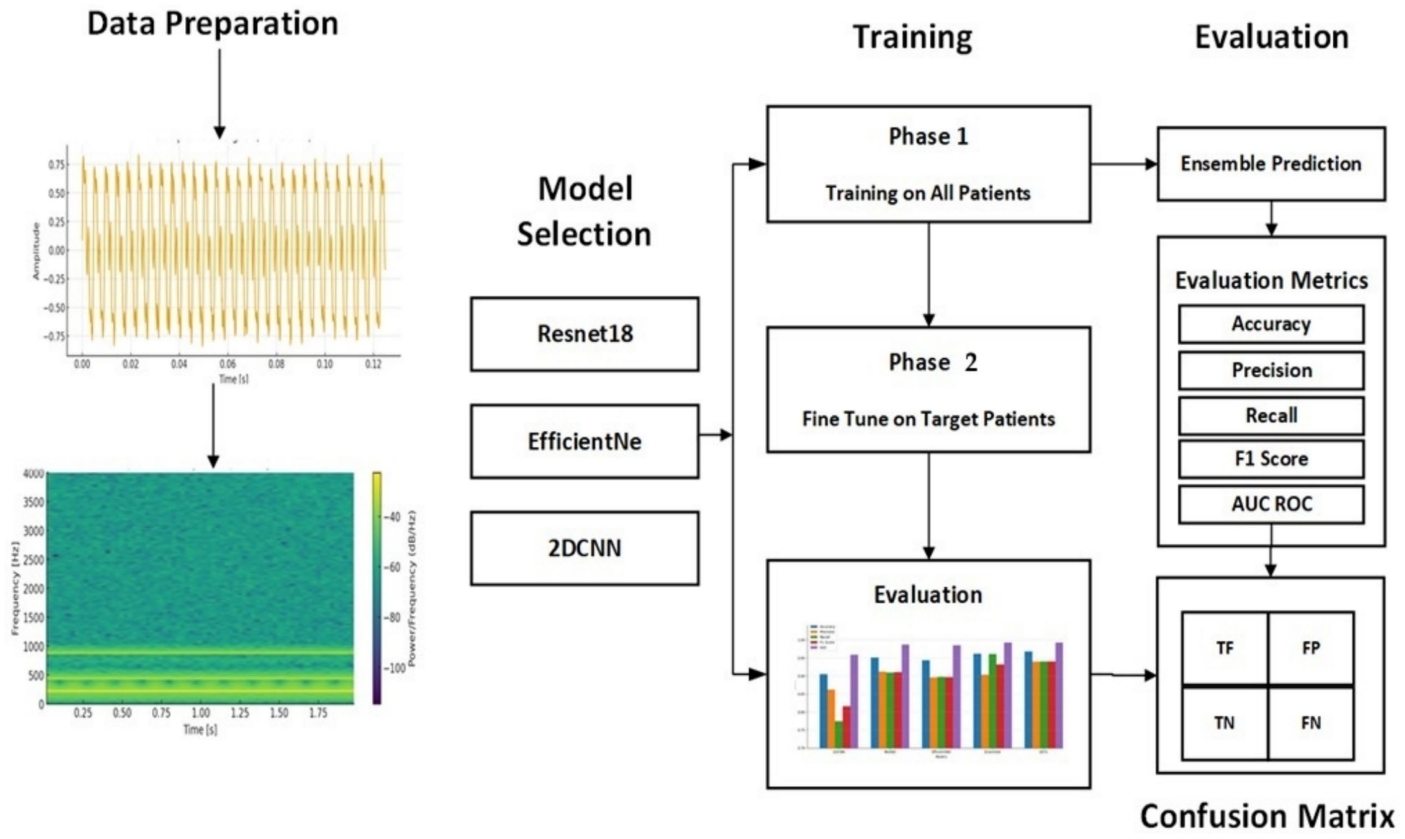
EDTL for personalized seizure detection.

A 2-Phase training model is proposed for personalized seizure detection. The steps involved can be seen in Figure 4. Raw EEG signals are passed to the model as input and converted in window segments. Each segment is then converted into a spectrogram using STFT. Single channel 2D images represented frequency vs. time are generated as output to be passed to CNN-based deep transfer learning models as input. Ensemble of deep transfer learning models is then applied following a 2-Phase training approach for personalized seizure detection. Phase 1 is the base training phase, where the models are trained on all the patients except the target patient. The goal is to learn the general transferable seizure patterns. Phase 2 is the fine-tuning or personalization phase, where the base models are fine-tuned on part of patient’s data. The target patient’s data is split into training and validation data where the base models are fine-tuned on the training data of the target patient. This fine-tuning allows the models to adapt to the patient-specific EEG patterns. The learning rate in the fine-tuning phase is kept lower than the base training phase to avoid the pre trained weights from being destroyed. After the fine-tuning phase, each patient has three models. Ensemble of the models is then applied final predictions.

#### 3.6.1 Phase-1: pre-trained model optimization for transfer learning

Each model was trained using appropriate optimization strategies to address challenges such as overfitting and vanishing gradients. Regularization techniques, dropout layers, early stopping, and learning rate scheduling were applied. Transfer learning was employed for ResNet-18 and EfficientNet-B0, allowing faster convergence with limited data, while the custom 2D-CNN was optimized through architecture tuning tailored to seizure spectrograms.

Two pre trained models and a customized 2DCNN model were trained on the processed data. The ResNet model (Yang et al., 2025), has been widely utilized in medical image processing applications (Haq et al., 2023). ResNet is a CNN-based architecture that utilizes residual connections to effectively train very deep networks by alleviating the network degradation and vanishing gradient problems encountered in traditional neural networks. Residual blocks are formed by using the skip connections, where some layers are skipped while activating the connections of layers to further layers in the network. Stacking of the residual blocks then constitutes the ResNet. ResNet prefers to learn fitting to residual mapping rather than underlying mapping. The ResNet18 variant of the ResNet network has been utilized in this study as one of the pre trained models in the EDTL models for seizure detection. Due to its relatively lightweight architecture, consisting only of 18 layers, compared to the deeper variants like ResNet-50 and ResNet-101, the number of trainable parameters is significantly reduced. The RenNet18 network has been widely utilized in various fields. Despite its reduced architecture, ResNet18 maintains powerful feature extraction capabilities balanced with computational efficiency. Regularization is used to skip the layers that degrade the performance of the network. The deep network model is degenerated into a shallow network by applying a constant mapping function Equation 3, thus avoiding the gradient explosion problem. The residual module, denoted by *H*(*x*) in the residual network, given the input *x*, is computed as:


H(x)=F(x)+x (3)

where *F*(*x*) is the output of the nonlinear transformation function applied to the input *x* through a sequence of two convolutional layers. That is, $F(x)=BN_{2}(\mathrm{Con}v_{2}(\mathrm{ReL}U_{1}(BN_{1}(\mathrm{Con}v_{1}(x)))))$ .

The gradient of H(x) is computed using the formula in Equation 4:


H′(x)=F′(x)+1 (4)

The above formula shows that, no matter how small the gradient *F′*(*x*) becomes, the total gradient value, represented by *H'*(*x*), will always be equivalent to a minimum of 1, due to the addition of 1 to the gradient value of F(x) . This is a crucial property of the ResNet model since this prevents the gradient from going too small during its propagation through the layers, thus effectively solving the gradient vanishing problem. The problem is caused by the increasing depth of the network in deep neural networks without residual connections. The second pre trained model used was EfficientNet (Uddin et al., 2024). EfficientNet is also a CNN-based architecture designed for performance and efficiency. The model utilizes compound scaling methods to scale the dimensions of width, depth, and resolution uniformly by using a compound coefficient. The lightweight EfficientNet-B0 variant is used in this work. We also utilized a customized two-dimensional convolutional neural model (2DCNN). The model consists of multiple layers designed to optimize feature extraction. The models were trained and optimized separately, utilizing the transfer learning capabilities of the pre trained models.

#### 3.6.2 Phase-2: ensemble of deep transfer learning

The three optimized models were stacked together to form an ensemble of a deep transfer learning method. The models are stacked to improve the overall prediction accuracy by aggregating the predictions from the three pre trained models. Each of the pre trained models learns to focus on different features or representations of input data. The combined output of the EDTL models thus significantly improves the predicted performance. Logistic regression is used as a meta-model to combine the predictions of the pre trained models to make the final decision. The proposed method combines the strengths of general-purpose pre trained models with the domain-specific custom 2DCNN model. The method combines the unique strengths of the individual models to leverage their complementary feature extraction capabilities. The ResNet model utilizes residual connections to learn deep hierarchical features, which can be particularly useful in domains with complex feature extraction requirements, such as spectrogram-based seizure signals, where it is important to capture long-range dependencies. EfficientNet enhances the efficiency of the EDTL by balancing computational efficiency and performance by scaling depth, width, and resolution. The custom 2DCNN is tailored to the domain specific features of seizure spectrograms and time-frequency representations. It utilizes domain specific architecture optimization by applying specialized convolution filters for capturing seizure patterns.

**Figure 5:**
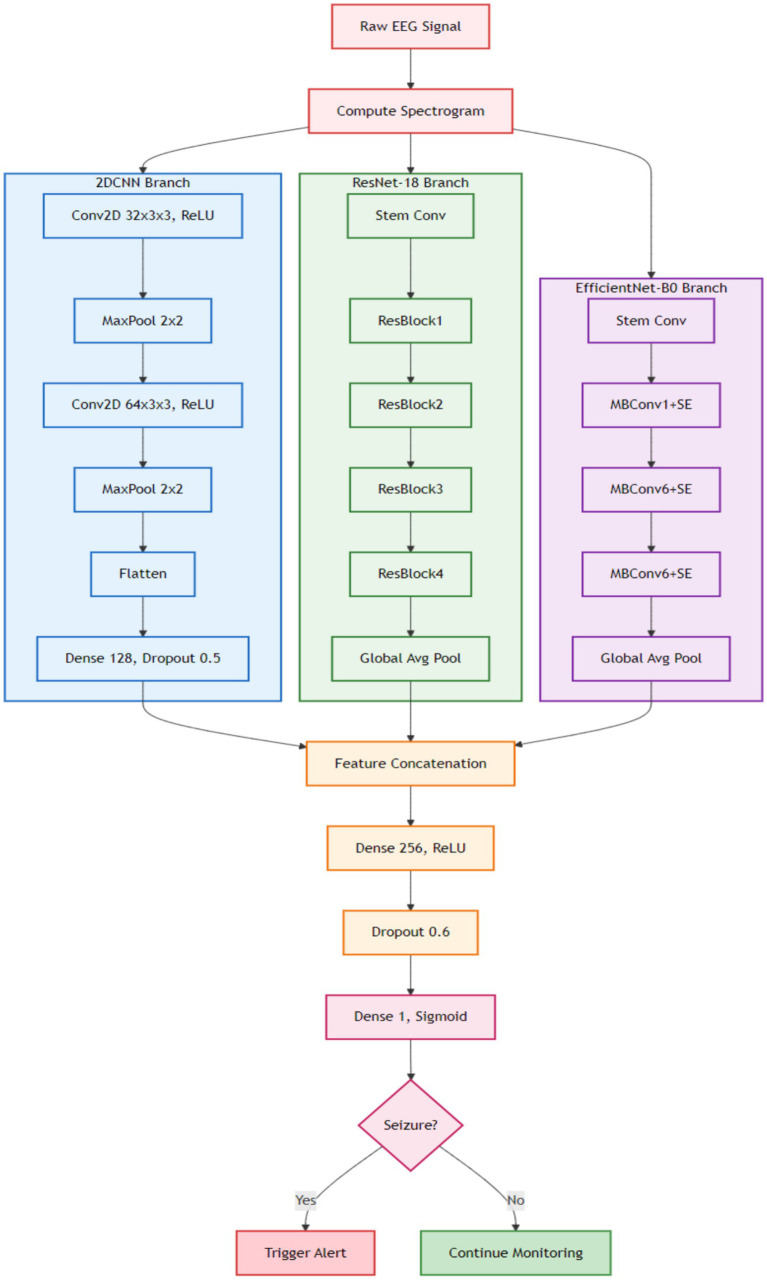
Architecture of the proposed EDTL model.

Raw EEG signals are transformed into spectrograms and are separately fed into each model, where each model independently extracts its own feature representation. The features extracted from the spectrograms are concatenated and passed to the next layer. Final predictions are then performed using the meta-model. The detailed structure of the proposed efficient model highlights the key components contributing to its improved performance, which can be seen in Figure 5.

To address potential training challenges such as vanishing gradient and overfitting, multiple strategies were employed. Residual connections in ResNet-18 and batch normalization in EfficientNet-B0 stabilized gradient flow, while ReLU activations in the 2DCNN further mitigated gradient vanishing. To reduce overfitting, dropout and L2 weight regularization were applied in addition to data augmentation. Early stopping and learning rate scheduling were also utilized to avoid over-training and improve model generalization.

## 4 Results

### 4.1 Description of the dataset

Two real world datasets were used to evaluate the performance of the proposed model. The details of the datasets are explained below.

#### 4.1.1 CHB-MIT scalp EEG database

CHB-MIT Scalp EEG database made publicly available by Shoeb (2010) was used to evaluate the performance of the proposed EDTL. The database contains EEG recordings of pediatric patients with intractable seizures. The recordings consist of 23 cases recorded from 22 subjects. Five of the subjects were males with ages between 3 and 22, while 17 subjects were females, with ages between 1.5 and 19. Recordings for one subject were repeated after a gap of 1.5 years. The recordings were conducted post anti-seizure medication withdrawal during their monitoring for possible surgical intervention and assessment of their seizure characteristics, at the Children’s Hospital Boston. There are a total number of 664 .edf files in the dataset, with each case containing 9–42 .edf files for each subject. One hundred twenty-nine of the files are records of seizures. The dataset also contains the subject information. One hundred ninety-eight of the records include seizures and are annotated accordingly. The protected health information and dates in the original files have been anonymized with surrogate details without disturbing the time relationships between the individual files.

#### 4.1.2 Turkish epilepsy EEG dataset

The Turkish Epilepsy EEG Dataset was released by Tasci et al. (2023) and is publicly available. The dataset consists of 71 healthy control signals and 50 seizure signals with a sampling frequency of 500 Hz.

### 4.2 Hyper parameters tuning

ResNet-18 architecture was used with transfer learning enabled, allowing the model to benefit from general image features, even when applied to spectrogram-based data. The input channels are modified to adapt the ResNet-18 for 1-channel data while retaining the architecture’s spatial feature extraction capacity. The classification head is also modified for binary classification. It reduces the dimensionality of the output features from ResNet’s last convolutional block. Fully connected 256-dimensional hidden layer is used to add representational capacity, allowing the network to learn higher level abstractions from the features extracted by the convolutional backbone. This transformation acts as a feature bottleneck, helping the model to prioritize the most informative components, such as seizures in EEG signals, while suppressing the less relevant information. To enable the model to separate data classes in high-dimensional feature spaces by learning non-linear, complex decision boundaries, Rectified Linear Unit (ReLU), a non-linear activation function, is applied. The model randomly drops 25% of the neurons during training. This acts as a regularize, mitigating the risk of overfitting—especially important when training on medical datasets like EEG data. Sigmoid activation function converts the single output logit into a probability in the range [0, 1], allowing the output to be interpreted as the likelihood of a seizure event. Similar modifications also applied for EfficientNet to adapt the model for processing single-channel EEG spectrogram and suiting to binary classification. The custom 2DCNN model is composed of six sequential convolutional layers, to extract hierarchical features from the input data, followed by an adaptive pooling layer, to adjust the special dimensions to a fixed size. Finally, fully connected layers are added to produce final predictions. The details of hyper parameters can be found in Table 1.

**Table 1:** Hyper parameters for custom 2DCNN, ResNet-18, and EfficientNet-B0.

Component	2DCNN	ResNet-18	EfficientNet-B0
Input channels	1 (EEG spectrogram)	1 (modified from RGB)	1 (modified from RGB)
Pre trained weights	No	Yes (ImageNet)	Yes (ImageNet)
Initial conv layer	Conv2d [1, 64, kernel_size = (2.4), padding = (1.2)]	Conv2d (1, 64, kernel_size = 7, stride = 2, padding = 3)	Conv2d (1, 32, kernel_size = 3, stride = 2, padding = 1)
Intermediate convs	5 more conv layers with increasing channels (up to 256)	ResNet-18’s default residual blocks	EfficientNet-B0’s compound scaling blocks
Pooling	MaxPool2d, AdaptiveAvgPool2d	MaxPooling, GlobalAvgPool	Mobile Inverted Bottleneck + GlobalAvgPool
Fully connected layers	256 → 128 → 64 → 1 + Sigmoid	512 → 256 → 1 + Sigmoid	1,280 → 1 + Sigmoid (via classifier)
Dropout	Two Dropouts (*p* = 0.25)	One Dropout (*p* = 0.25)	One Dropout (*p* = 0.2)
Activation	ReLU + Sigmoid	ReLU + Sigmoid	Swish (internally) + Sigmoid
Final activation	Sigmoid() (for BCELoss)	Sigmoid() (for BCELoss)	Sigmoid() (for BCELoss)
Architecture type	Fully custom convolutional architecture	Residual network with skip connections	Compound-scaled efficient architecture

### 4.3 Optimization of the models

**Figure 6:**
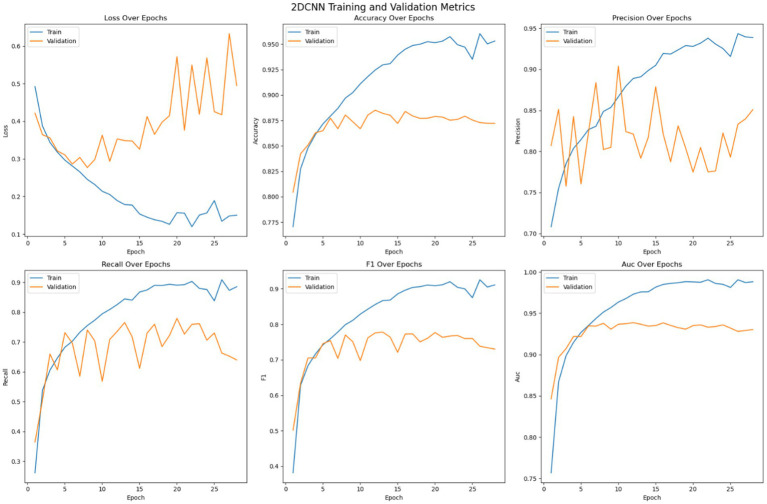
Optimization of the 2DCNN model.

Each of the pre trained model and the customized 2DCNN were optimized independently before being stacked for transfer learning. The models were trained on the dataset over 200 epochs with early stopping enabled with a patience value of 20. Figure 6 shows the optimization of the 2DCNN model on the given dataset. The loss error, precision, recall, accuracy, F1 measure and AUC have been reported for the training and validation set. The figure shows that the model stabilizes over the iterations and achieves early convergence and shows tendencies to over fit afterwards. Initially, the loss on both the training data and validation data decreases while the accuracy shows consistent improvement, where the training accuracy reaches over 96% and the validation accuracy crossing 88%. Then the model tends to over fit with validation error not improving over the epoch, while the accuracy of the model stabilizes. Early stopping is used to avoid overfitting of the model. Precision performance of the model on training data shows steady improvement, reaching up to 93%, while the validation precision shows variation with the best value reaching up to 90%. Recall metrics of the model shows consistency with training recall reaching up to 90% and the validation recall achieving a maximum value of 78%. The F1-measure of the model remains consistent for both the training and validation sets, with the training F1 score crossing 91% and validation F1 score reaching up to 78%. The AUC performance measure shows consistency over all the epochs. The AUC score for the training set of the model reaches up to 98% and for the validation set the score achieves a maximum value of 94%. Model with best validation loss is saved to be further used as part of the ensemble learning.

**Figure 7:**
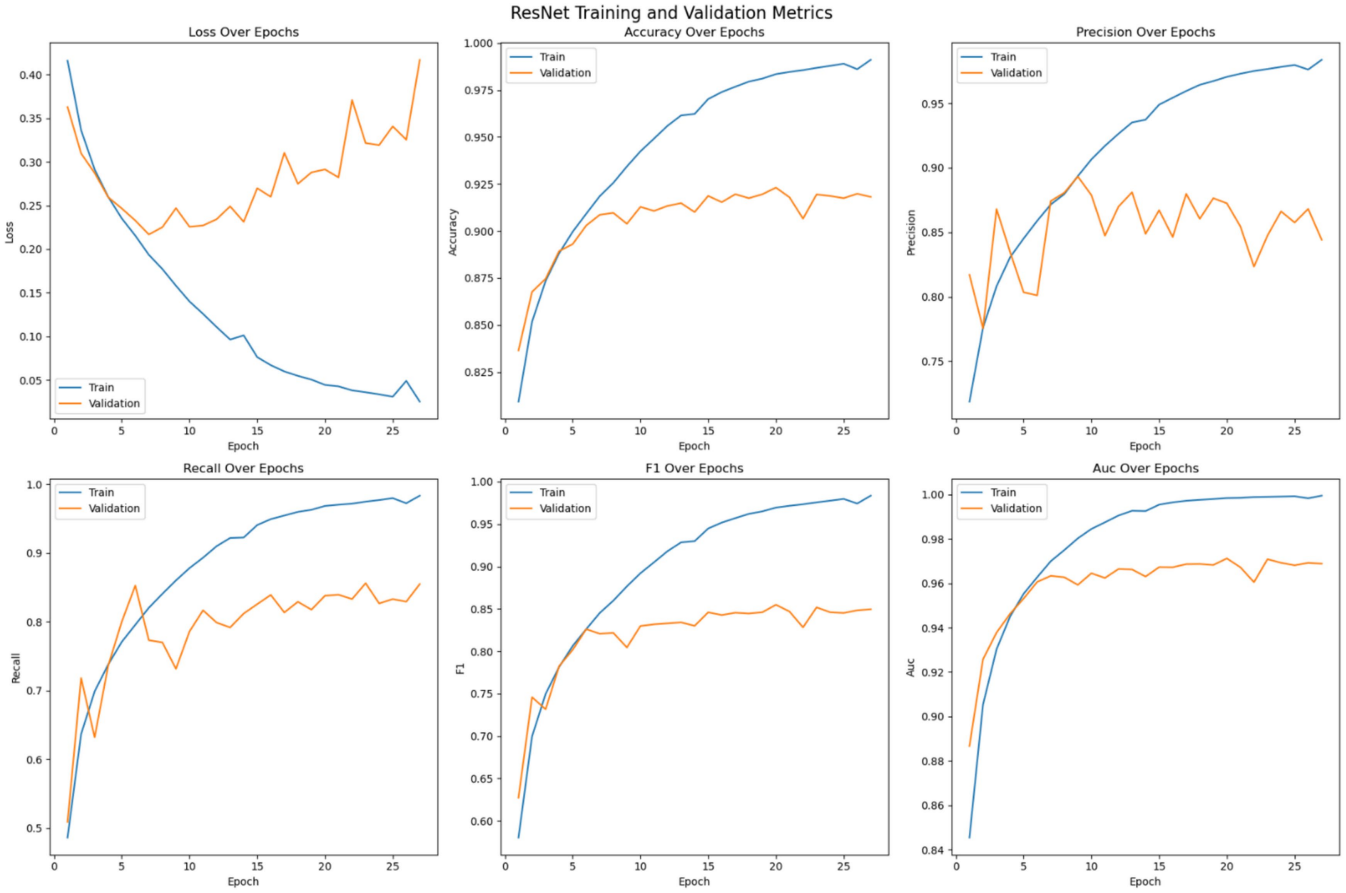
Optimization of the ResNet model.

Figure 7 shows the optimization of the pre trained ResNet model on the given dataset. The loss error, precision, recall, accuracy, F1 measure and AUC have been reported for the training and validation set. As shown in the Figure, like the customized 2DCNN model, the ResNet model stabilizes over the iterations and achieves early convergence. However, the model shows tendencies to over fit afterwards, with widening difference between the training and validation loss. Initially, both the training and validation loss decrease while the accuracy shows consistent improvement, with the training accuracy reaching over 98% and the validation accuracy crossing 90%. Then the model tends to over fit with validation error not improving, or getting worse, over the subsequent epochs, while the accuracy of the model stabilizes. Early stopping is used to avoid overfitting of the model. Precision of the model on training data shows steady improvement, reaching up to 98%, while the validation precision shows comparatively low variation with the best value reaching up to 90%. Recall metrics of the model shows continued improvement on the training data, reaching up to 98% and the validation recall achieving a maximum value of 84%. The F1-measure of the model remains consistent for both the training and validation sets, with the training F1 score crossing 98% and validation F1 score reaching up to 84%. The AUC performance measure shows consistency over all the epochs. The AUC score for the training set of the model reaches up to 100% and for the validation set the score stabilizes at a maximum value of 96%. Model with best validation loss is saved to be further used as part of the ensemble learning.

**Figure 8:**
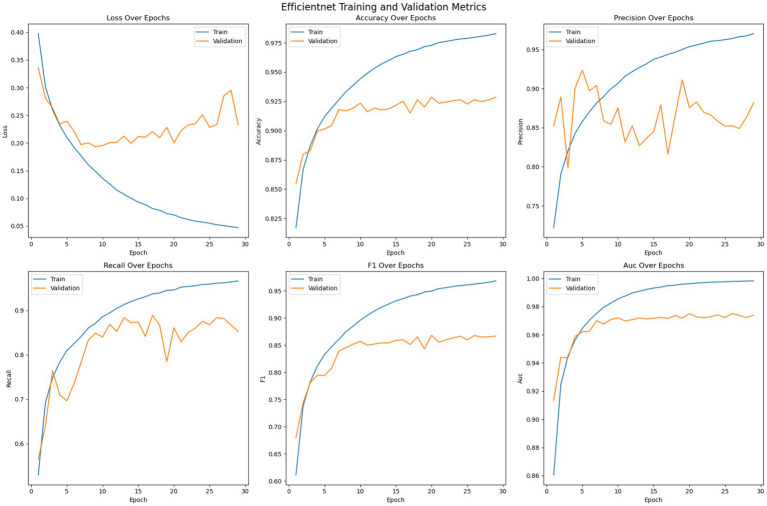
Optimization of the EfficientNet model.

Figure 8 shows the optimization of the pre trained EfficientNet model on the given dataset. The loss error, precision, recall, accuracy, F1 measure and AUC have been reported for the training and validation set. The figure suggests that, unlike the customized 2DCNN and ResNet models, the EfficientNet model converges over more iterations and shows comparatively stable performance. The model shows less tendencies to over fit, with comparatively lower gaps between the training and validation loss. Moreover, the validation loss is stable over epochs as compared to other models, where the performance on the validation set decreases after certain iteration, indicating overfitting. Initially, both the training and validation loss decrease while the accuracy shows consistent improvement, with the training accuracy reaching up to 98% and the validation accuracy crossing 92%. Then the model generalizes better to the validation set across the validation metrics, with validation error showing improvement, and stabilizing over the subsequent epochs, while the accuracy of the model stabilizes. Again, early stopping is employed to avoid overfitting of the model. Precision performance of the model on training data shows steady improvement, reaching up to 98%, while the validation precision shows comparatively low variation with the best value reaching up to 90%. Recall metrics of the model shows continued improvement on the training data, reaching up to 98% and.

**Figure 9:**
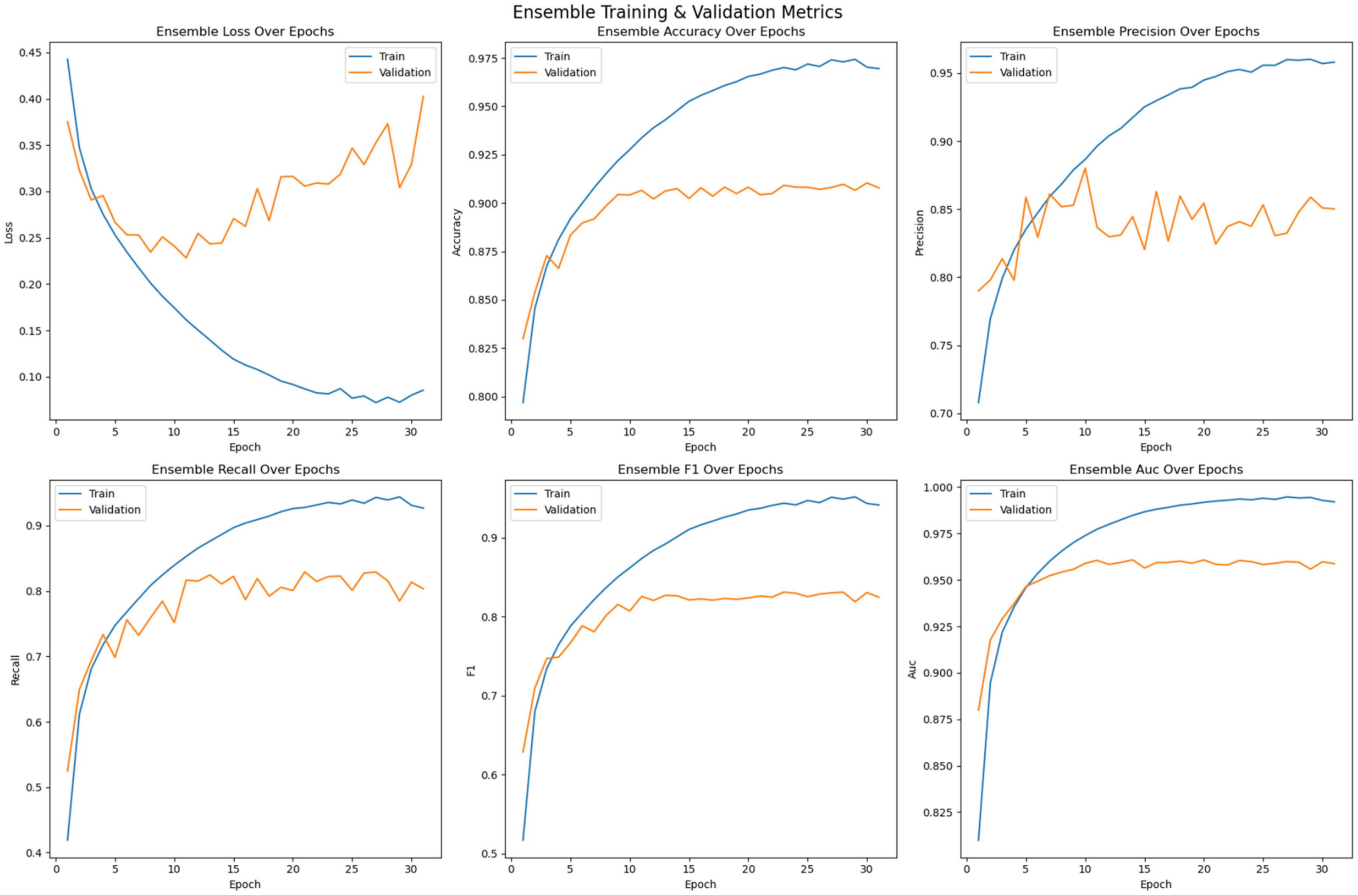
Ensemble (average) training and validation.

The EDTL model is an ensemble of 2DCNN, ResNet-18, and EfficientNet-B0, where features from each base model are extracted and concatenated for final classification. Unlike individual neural networks, the ensemble does not update weights during training; it only combines the predictions of the pre-trained base models. Therefore, epoch-wise training or validation loss/accuracy curves are not available for EDTL. Its performance is reported using final evaluation metrics (Accuracy, Precision, Recall, F1-score, AUC) on the test set. However, ensemble evaluation curves can be simulated epoch by averaging the evaluation metrics across the individual models, shown in Figure 9. Since each model might converge at the different number of epochs, the model with maximum number of epochs is identified and the missing epochs for the other models are padded with the last value to run the ensemble curves for as long as the longest trained model.

### 4.4 Performance evaluation on the test data

**Figure 10:**
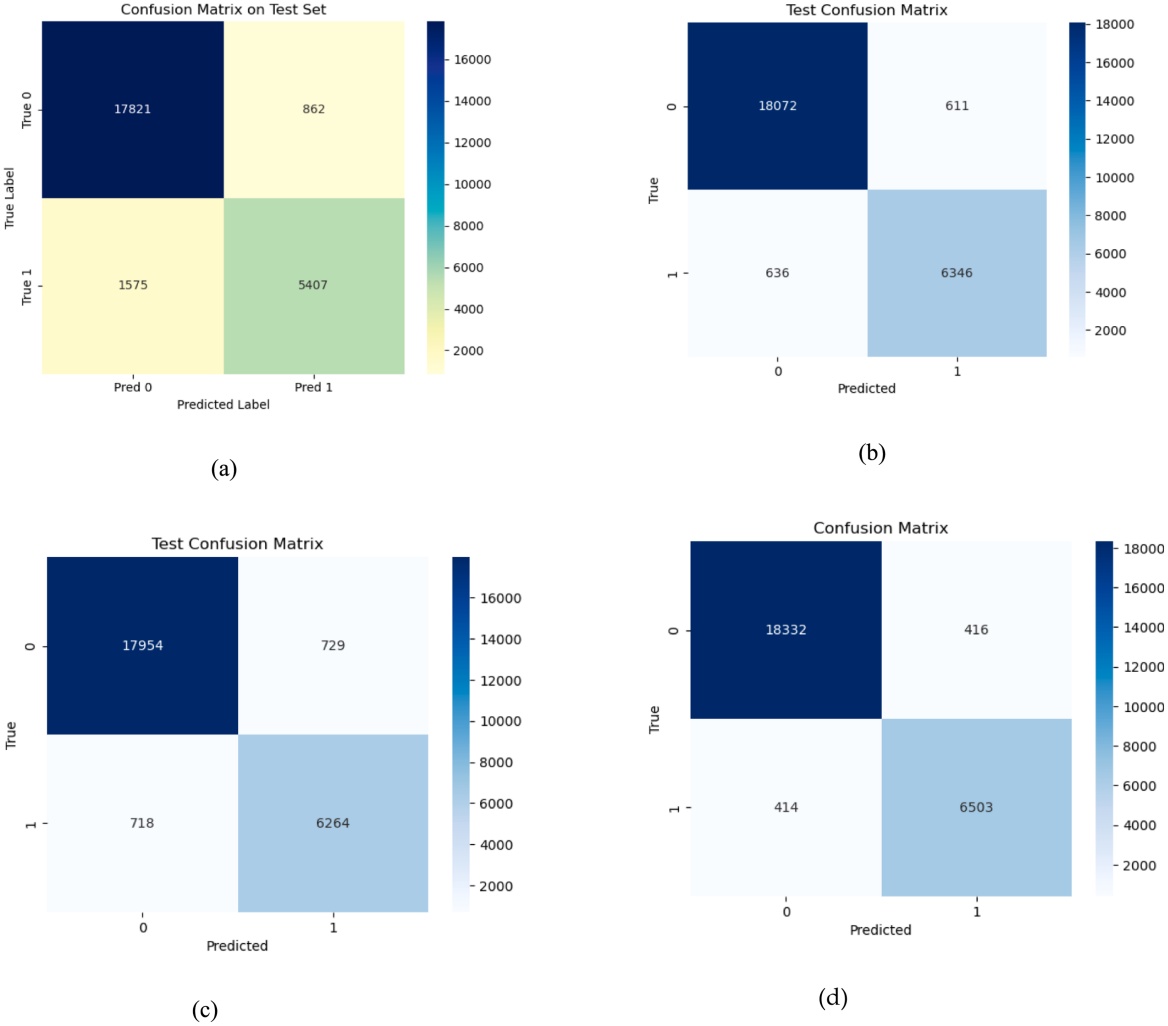
Confusion matrices for the models on test data; **(a)** for 2DCNN; **(b)** for ResNet18; **(c)** for EfficientNet-B0; **(d)** for EDTL.

The proposed EDTL mechanism is evaluated and compared against the optimized DL models. 15% of the dataset is used as a test set to evaluate the performance of the model on unseen data. The optimized models are comparatively evaluated on the test data, and the proposed EDTL method shows considerable improvement over the individual pre trained DL models. The confusion matrices of the models can be seen in Figure 10a, depicts the confusion matrix of 2DCNN model on the test data. The model correctly classifies 17,821 samples into non-seizure and 5,407 samples into seizure classes. The model incorrectly classifies 862 samples into non-seizure and 1,575 samples into seizure classes. Figure 10b depicts the confusion matrix for the ResNet method. The model accurately classifies 18,072 samples into non-seizure and 6,346 samples into seizure classes. The model inaccurately classifies 611 samples into non-seizure class and 636 samples into seizure class, showing an improved performance. Figure 10c depicts the confusion matrix for the EfficientNet method. The model accurately classifies 17,954 samples into non-seizure class and 6,264 samples into seizure class and inaccurately classifies 729 samples into non-seizure and 718 samples into seizure class. The confusion matrix of the EDTL for the test data is depicted in Figure 10d. The model significantly outperforms the deep transfer and custom models. It correctly classifies 18,332 samples into non-seizure and 6,503 samples into seizure classes. We can observe the inaccuracy of the model with the classification of 416 seizure samples into non-seizure class and 414 non-seizure samples into seizure class.

Table 2 compares the performance of EDTL on the most frequently used evaluation metrics in the literature with the standard deep transfer and customized CNN models. The model shows significant performance improvement over most of the metrics. The best scores have been highlighted.

**Table 2:** Comparison of the models’ performance.

Model	Accuracy	Precision	Recall	F1 Score	AUC
ResNet	95.73%	90.64%	**94%**	92.29%	98.97%
EfficientNet	94.61%	89.31%	91.11%	90.20%	98.52%
2DCNN	91.54%	87.40%	80.54%	83.83%	96.71%
EDTL (Ensemble)	**96.65%**	**94.07%**	93.57%	**93.82%**	**99.23%**

Bold values mean high performance of the EDTL (Ensemble) model.

**Figure 11:**
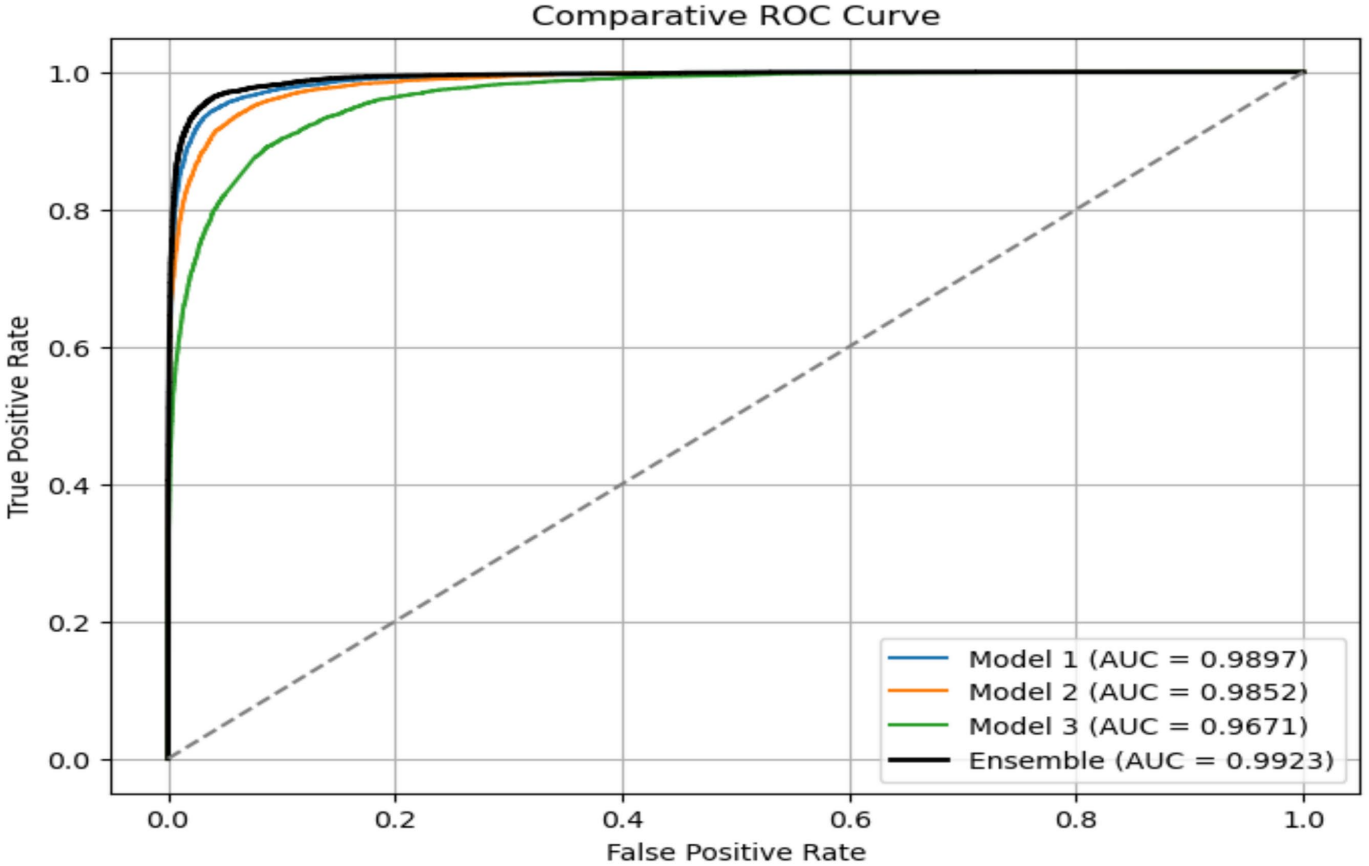
Performance on ROC curve of the EDTL compared with other DL models.

**Figure 12:**
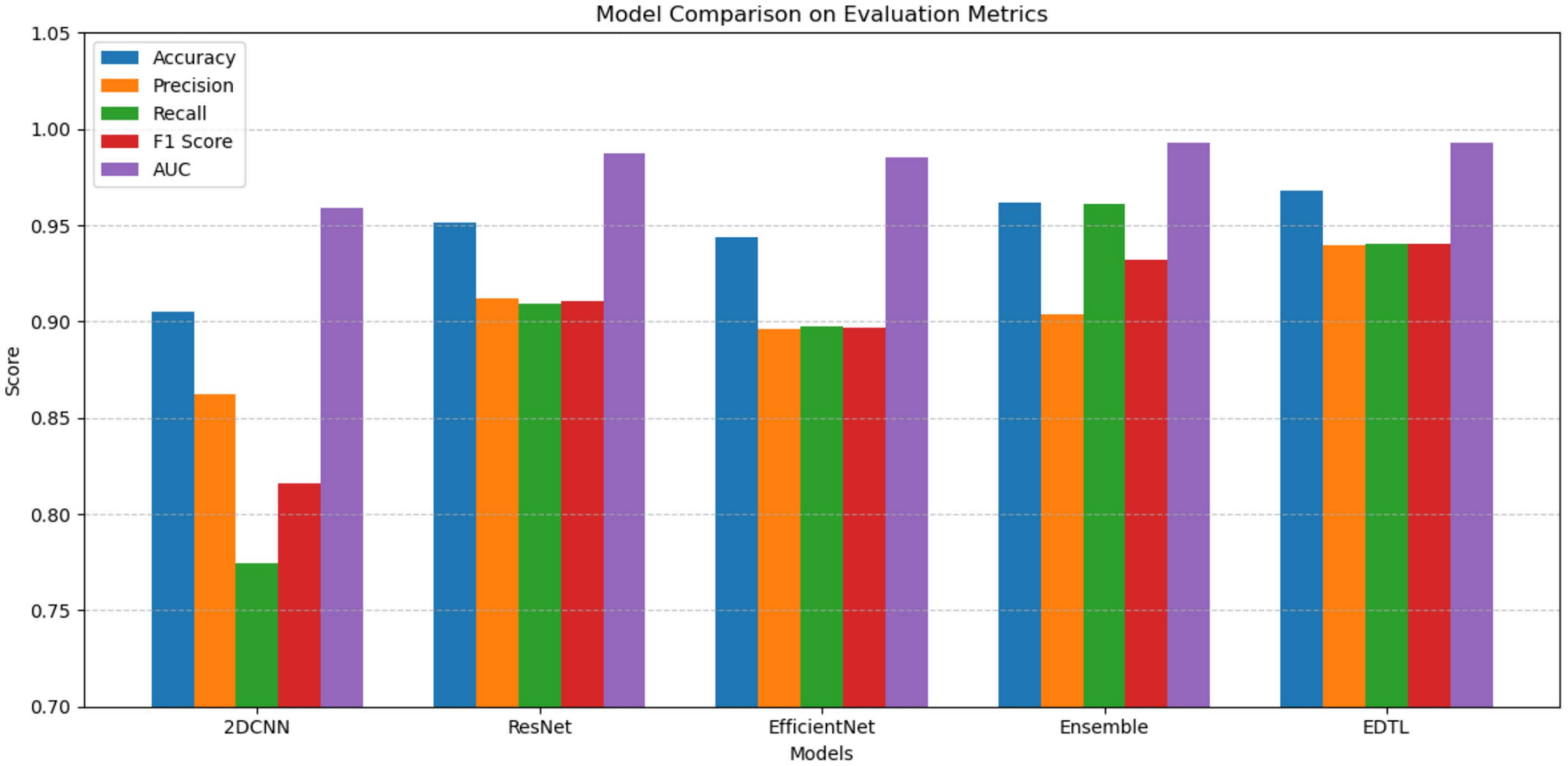
Comparison of the EDTL performance with DL models.

The EDTL model achieves a score of 96.65% for accuracy, score of 94.07% for precision, a recall score of 93.57%, a score of 93.82% for F1, and an AUC score of 99.23%. The performance of the EDTL compared to the other models suggests considerable improvement without increasing the complexity of the model. Figure 11 presents a comparative analysis of the AUC values obtained from the evaluated models. In Figure 11, Model 1 displays the ResNet18 method, Model 2 shows the EfficientNet-B0, and Model 3 shows the 2DCNN model. The proposed EDTL framework is shown by the Ensemble mechanism. The proposed framework significantly outperforms the other models on the AUC score, achieving a score of 99.23%. The ResNet method performs second best with an AUC score of 98.97%, followed by the EfficientNet method with a score of 98.52%. Although the customized 2DCNN shows good performance with an AUC score of 96.71%, the deep transfer learning-based models significantly outperform the model, with the proposed EDTL performing better overall than the other models, as shown in Figure 12.

**Figure 13:**
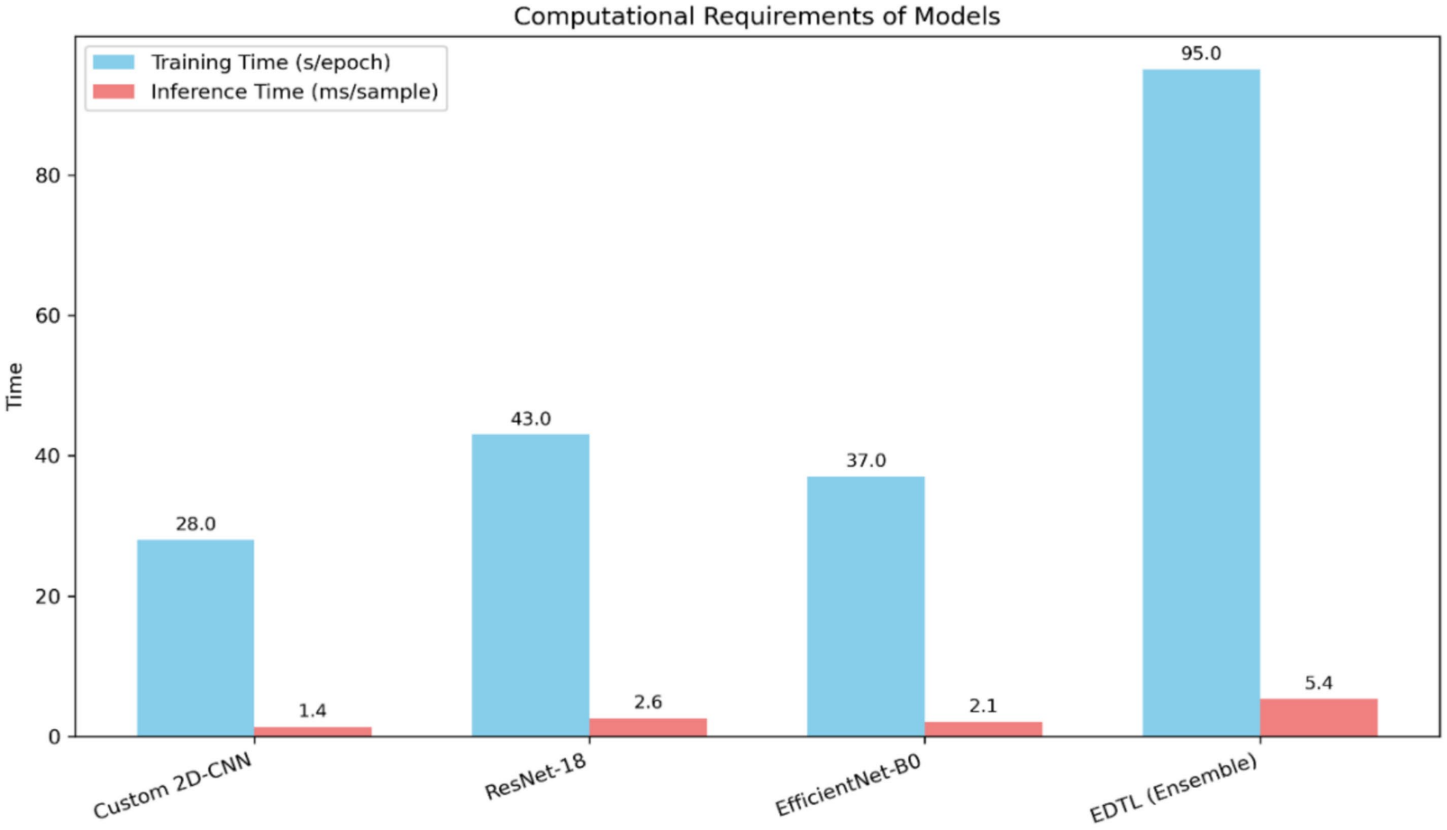
Computational requirements of the proposed models.

To further analyze the efficiency of the proposed methodology, the computational requirements of individual models and the ensemble were measured in terms of training time per epoch and inference time per sample, as illustrated in Figure 13. The figure compares training time per epoch (in seconds) and inference time per sample (in milliseconds) for Custom 2DCNN, ResNet-18, EfficientNet-B0, and the proposed ensemble (EDTL). The ensemble requires more computation but delivers superior performance and robustness across datasets.

Table 3 shows how our model compares with other studies that used the same CHB-MIT EEG dataset. The comparison has been performed with results reported in the original works or the relevant literature. Some models, such as LSTM with handcrafted features, gave high accuracy (98.43%), while others, like 3D CNN and Vision Transformers, had lower results, with accuracy around 80–85%. The EDTL model performed very well, with 96.65% accuracy, 94.07% sensitivity, and a high AUC of 99.23%. This shows our model is strong, balanced, and works better than many existing methodologies.

**Table 3:** Comparison of proposed method with SOTA on the CHB-MIT EEG dataset.

Study	Model	Data	Accuracy	Sensitivity	Specificity	AUC
Hu et al. (2020) and Taherinavid et al. (2024)	Bi LSTM	Raw EEG	—	93.61%	91.85%	
Cao et al. (2025) and Pielawski and Wählby (2020)	LSTM	Handcrafted features	98.43%	97.84%	99.21%	—
Wang et al. (2021) and He et al. (2016)	3D CNN	Raw EEG	80.5%	85.8%	75.1%	—
Deng et al. (2023) and Xu et al. (2023)	HViT-DUL	Raw EEG	—	87.9	—	
Ozcan and Erturk (2019) and Tan and Le (2019)	3D CNN	Raw EEG	—	85.71	—	0.096
Godoy et al. (2022) and Shoeb (2010)	Temporal multi-channel vision transformer	Raw EEG	82.0	80.0		
Proposed	EDTL (Ensemble)	CHB-MIT Scalp EEG	96.65%	94.07%	93.57%	99.23%
	EDTL (Stacking Ensemble)	Turkish Epilepsy EEG	98.07%	97.69%	97.97%	99.77%
	EDTL (Ensemble)	Turkish Epilepsy EEG	92.66%	83.98%	91.08%	99.72%

Compared to previous studies, the proposed ensemble models showed clear improvements in performance. Earlier works, such as those by Taherinavid et al. (2024), Wang et al. (2021), He et al. (2016), and Godoy et al. (2022), and Shoeb (2010) achieved moderate accuracy and sensitivity, while methods relying on handcrafted features, such as Cao et al. (2025) and Pielawski and Wählby (2020), performed strongly but were limited by feature engineering requirements. The slightly lower performance of our proposed methodology on the CHB-MIT Scalp EEG dataset compared to Cao et al. (2025), Pielawski and Wählby, (2020) (96.65% vs. 98.43%) may be due to differences in preprocessing and feature engineering. Cao et al. relied on handcrafted features with an LSTM model, whereas our method uses an ensemble-based approach without handcrafted features.

However, when applied to the Turkish Epilepsy EEG dataset, our methodology achieved competitive or even higher results (e.g., 98.07% accuracy with stacking ensemble and 99.77% specificity), which highlights the robustness and generalizability of the proposed approach across different datasets. In contrast, the proposed EDTL and stacking ensemble models demonstrated consistently high accuracy, sensitivity, specificity, and AUC across both the CHB-MIT and Turkish Epilepsy EEG datasets. This highlights their robustness and reliability, indicating that ensemble-based approaches are more effective for epilepsy detection compared to single DL models used in earlier studies.

## 5 Discussion

Approximately 1% of the global population is affected by epilepsy, representing millions of individuals worldwide who could benefit from improved seizure detection and prediction technologies. The development of advanced computational methods for detecting seizures has garnered significant attention over the past several years, motivated by their potential to significantly improve the quality of life for individuals affected by epilepsy through more accurate, timely, and automated intervention strategies. A comprehensive, computationally efficient framework for personalized seizure detection is presented in this work, performing rigorous analysis of pre trained models, augmenting the pre trained models and domain-specific optimization. Empirical evaluation of the proposed approach is performed, demonstrating its advantages in terms efficiency and flexibility, without compromising the performance. EDTL is efficient because it uses pre trained networks, reducing training time and computational costs. It is also flexible, as it performs well on raw EEG data from different patients without needing complex feature extraction. The proposed EDTL framework represents a sophisticated approach to personalized seizure detection that addresses the intrinsically variable nature of seizure patterns across individual patients. The methodology combines the strengths of established deep learning architectures—ResNet and EfficientNet—with a customized 2DCNN specifically designed for this application.

The preprocessing pipeline demonstrates careful consideration of the unique characteristics of EEG data. Raw recordings from seizure patients undergo transformation into standardized EEG signals, followed by the application of personalized sliding windows to extract spectrograms tailored to individual patients. This patient-specific approach is crucial given the significant inter-patient variability in brain structure, seizure patterns, and signal characteristics. The normalization of EEG signals ensures consistent scaling across different recordings, while the STFT enables continuous analysis of signals over short time intervals, capturing the dynamic nature of seizure activity.

The training strategy employed demonstrates a methodical approach to model optimization. Each component of the ensemble—the pre trained models and the customized 2DCNN—were optimized independently by over 200 epochs with early stopping mechanisms to prevent overfitting. The patience value of 20 epochs provided sufficient opportunity for model convergence while maintaining computational efficiency. The customized 2DCNN model exhibited typical DL training characteristics, with both training and validation losses decreasing initially while accuracy improved consistently. Training accuracy reached over 96% with validation accuracy exceeding 88%. However, the model showed tendencies toward overfitting as training progressed, with validation metrics stabilizing while training metrics continued to improve. The precision reached 93% on training data and 90% on validation data, while recall achieved 90 and 78%, respectively. The F1-measure maintained consistency across both sets, with training F1 crossing 91% and validation F1 reaching 78%. The AUC performance demonstrated strong discriminative ability, achieving 98% on training data and 94% on validation data. The ResNet model demonstrated superior performance characteristics compared to the custom 2DCNN, achieving over 98% training accuracy and 90% validation accuracy. This pertained model showed excellent precision and recall metrics, with training values reaching 98% for both measures and validation precision and recall achieving 90 and 84%, respectively. The F1-scores reflected this strong performance, with training F1 exceeding 98% and validation F1 reaching 84%. Notably, the AUC performance was exceptional, achieving 100% on training data and 96% on validation data, indicating excellent discriminative capability. EfficientNet displayed the most stable training characteristics among the three models, converging over more iterations but showing less tendency to over fit. The model achieved an accuracy of 98% on training data and 92% on validation data, representing the highest validation performance among the individual models. The precision and recall metrics were consistent with ResNet, reaching 98% on training data and 90 and 84% on validation data, respectively. The F1-scores mirrored these results, with training F1 crossing 98% and validation F1 reaching 84%. The AUC performance was strong at 99% for training and 97% for validation data. The confusion matrix analysis reveals the practical performance of each model in differentiating the seizure from non-seizure states. The custom 2DCNN correctly classified the majority of samples in both classes but showed higher misclassification rates compared to the pre trained models. ResNet demonstrated superior classification performance with higher true positives and true negatives across both training and validation datasets. EfficientNet showed balanced performance with good accuracy in both seizure and non-seizure classification.

The ensemble approach leveraged the complementary strengths of these individual models, achieving the highest overall performance of 99.23% on the AUC ROC curve. This superior performance demonstrates the value of combining different architectural approaches and optimization strategies in a unified framework.

The personalized nature of the proposed framework addresses a critical challenge in seizure detection—the significant variability in seizure manifestations across different patients. By incorporating patient-specific features and personalized sliding windows, the system can adapt to individual characteristics while maintaining high accuracy. The achievement of 99.23% AUC performance suggests that the system could provide clinically relevant seizure detection with minimal rates for both false positives and false negatives. The computational efficiency of the framework is enhanced using transfer learning, which reduces training time and computational requirements compared to training large models from scratch. The ensemble approach, while requiring multiple models, achieves superior performance that justifies the additional computational overhead.

### 5.1 Limitations and future directions

The study demonstrates the potential of ensemble deep transfer learning for personalized seizure detection. Despite the promising performance of the model, several considerations warrant further investigation. The generalizability of the approach across different EEG recording systems and patient populations requires validation. Additionally, the real-life implementation of the ensemble system in clinical settings would need to address computational constraints and response time requirements.

We used the CHB-MIT Scalp EEG dataset for evaluating the model. The dataset is large and complex with long-term recordings of EEG from multiple patients, making it suitable for evaluating personalized seizure detection. The framework’s reliance on high-quality EEG data and the need for patient-specific optimization may present challenges in resource-limited clinical environments. Due to the depth and complexity of this dataset, we focused our analysis on it. However, in the future, we intend to evaluate the generalization capability of the EDTL model by exploring additional datasets and compare it with results from other studies. Future work could explore methods to reduce the computational requirements while maintaining the high performance achieved by the current ensemble approach.

## 6 Conclusion

Epilepsy is diagnosed in millions of people (about 1% percent of the world’s population) as a common brain disease. The study and prediction, and detection of seizures can significantly improve the lives of epilepsy patients. The study has attracted vast attention over recent years, specifically involving advanced computation methods. This paper presents EDTL models for personalized seizure detection. The method combines ResNet and EfficientNet methods along with a customized 2DCNN method for patient specific seizure detection using EEG data. Raw data from the recordings of seizure patients is transformed into EEG signals. Personalized sliding windows are used to extract and store spectrograms for the patients. Patient specific features are extracted from individual records. EEG signals are normalized for consistent scaling. STFT is then applied for continuous window slicing over short time intervals. The transformed data is then passed on to train and optimize the models independently and later combined into EDTL. A comparative evaluation is performed using standard evaluation metrics. The performance of the individual method is compared with the proposed EDTL, with the EDTL having the highest performance of 99.23% on the AUC ROC curve. The ensemble of pre trained models along with the customized CNN based models with domain specific optimization ensures that optimum results are obtained without compromising the efficiency of computation.

## Data Availability

The datasets presented in this study can be found in online repositories. The names of the repository/repositories and accession number(s) can be found at: CHB-MIT: https://physionet.org/content/chbmit/1.0.0/, Turkish EEG: https://www.kaggle.com/datasets/buraktaci/turkish-epilepsy.
